# Sporadic pancreatic neuroendocrine neoplasms: A retrospective clinicopathological and outcome analysis from a Latvian study group

**DOI:** 10.3389/fsurg.2023.1131333

**Published:** 2023-03-20

**Authors:** Margarita Ptasnuka, Arturs Truskovs, Arturs Ozolins, Zenons Narbuts, Maris Sperga, Haralds Plaudis

**Affiliations:** ^1^ Department of General and Emergency Surgery, Riga East Clinical University Hospital, Riga, Latvia; ^2^ Department of Surgery, Pauls Stradiņš Clinical University Hospital, Riga, Latvia; ^3^ Department of Surgery, Riga Stradiņš University, Riga, Latvia; ^4^ Department of Infectious Pathology, Pathology Center, Riga, Latvia

**Keywords:** pancreatic neuroendocrine neoplasm, surgery, postoperative complications, survival, epidemiology

## Abstract

**Background:**

Although pancreatic neuroendocrine neoplasms (PNEN) are rare, there has been a constant increase in incidence. Furthermore, PNEN present unique clinical behaviors and long-term survival can be expected even in the presence of metastases as compared with ductal adenocarcinoma of the pancreas. Determining the best therapeutic approach and proper timing of therapy requires knowledge of reliable prognostic factors. Therefore, the aim of this study was to explore clinicopathological features, treatment, and survival outcomes of patients with PNEN based on Latvian gastroenteropancreatic neuroendocrine neoplasm (GEP-NEN) registry data.

**Method:**

Patients with confirmed PNEN at Riga East Clinical University Hospital and Pauls Stradins Clinical University Hospital, between 2008 and 2020, were retrospectively analyzed. Data were collected and included in EUROCRINE, an open-label international endocrine surgical registry.

**Results:**

In total, 105 patients were included. The median age at diagnosis was 64 years (IQR 53.0–70.0) for males and 61 years (IQR 52.5–69.0) for females. In 77.1% of patients, tumors were hormonally nonfunctional. Among those with functioning PNEN, 10.5% of patients presented with hypoglycemia and were diagnosed with insulinoma, 6.7% of patients presented with symptoms related to carcinoid syndrome; 30.5% of patients showed distant metastases at the time of diagnosis, and surgery was performed in 67.6% of patients. Notably, for five patients with nonfunctional PNEN <2 cm, a “watch and wait” approach was used; none of the patients developed metastatic disease. The median length of hospital stay was 8 days (IQR 5–13). Major postoperative complications were found in 7.0% of patients, and reoperation was conducted for 4.2% of patients, due to postpancreatectomy bleeding (2/71) and abdominal collection (1/71). The median follow-up period was 34 months (IQR 15.0–68.8). The OS at the last follow-up was 75.2% (79/105). The observed 1-, 5- and 10-year survival rates were 87.0, 71.2 and 58.0, respectively. Seven of the surgically treated patients had tumor recurrence. The median time of recurrence was 39 months (IQR 19.0–95.0). A univariable Cox proportional hazard analysis provided evidence that a nonfunctional tumor, a larger tumor size, the presence of distant metastases, a higher tumor grade, and the tumor stage were strong, negative predictors of OS.

**Conclusion:**

Our study represents the general trends of clinicopathological features and treatment of PNEN in Latvia. For PNEN patients, tumor functionality, size, distant metastases, grade, and stage may be useful to predict OS and must be confirmed in further studies. Furthermore, a “surveillance” strategy might be safe for selected patients with small asymptomatic PNEN.

## 1. Introduction

PNEN are a rare subgroup of pancreatic tumors that account for 1%–2% of all pancreatic malignancies with a peak incidence between the fourth and the sixth decade of life (1, 2). Notably, in recent years, the reported incidence rate of PNEN has been dramatically increasing, perhaps as a result of the wide availability of cross-sectional imaging studies. In United States, the reported incidence from 2004 to 2012 increased from 0.4 per 100,000 to over 0.8 per 100,000 (3). In Latvia, PNEN accounted for 30.7% of all GEP-NEN between 2006 and 2018, as well the pancreas was the most frequent primary site of GEP-NEN (4).

An important feature of PNEN is clinical and biological heterogeneity among slow-growing localized tumors with an indolent behavior as well as aggressive neoplasms presenting with distant metastases. Considering this, over the last couple of decades, a number of WHO classification changes have occurred (5). A review of the literature has shown that up to 90% of PNEN are nonfunctional and without clinically relevant hormonal symptoms (6). Moreover, in recent years, sporadic nonfunctional PNEN have been more frequently discovered in patients undergoing diagnostic evaluation for unrelated conditions (7).

The management of patients with PNEN should be multidisciplinary. Complete surgical tumor removal remains to be the cornerstone of therapy for locoregional PNEN. Types of potentially curative surgery may include variable resection (e.g., Whipple procedure and distal pancreatectomy), parenchyma-sparing resection with or without regional lymphadenectomy, or even total pancreatectomy. Additionally, several studies have suggested that patients with sporadic, nonfunctional tumors less than 2 cm may be safely observed with nonoperative management (8–11). For selected patients with advanced PNEN, resection of both the primary tumor and liver metastases can be performed (12). The recommended options for patients with unresectable disease include treatment with somatostatin analogues, radiolabeled somatostatin analogs, or systemic targeted therapy with chemotherapy or ablative therapy (13).

Importantly, as compared with GEP-NEN, PNEN have the lowest 5-year survival rates (14). Many factors relate to the outcomes of PNEN. As shown in the literature, liver metastases play significant roles for lower survival in PNEN; the 5-year survival rate has been shown to be significantly worse than patients without liver metastasis (15). To date, many studies from all around the world have researched the relevant prognostic factors of PNEN affecting overall survival (1, 16, 17).

Only a few studies on PNEN in a large population are available, hence, the need for more detailed information on this rare pancreatic tumor type. We aimed to analyze the clinicopathological characteristics through our experience in treatment of PNEN at two high-volume specialized centers in Latvia.

## 2. Materials and methods

### 2.1. Patient identification and data source

This study was designed as an observational, retrospective, multicenter analysis that used a supplemented clinical database of Latvian GEP-NEN patients. All adult (≥18 years old) patients with a histologically confirmed diagnosis of PNEN between January 2008 and December 2020 and managed at Riga East Clinical University Hospital (RECUH) and Pauls Stradins Clinical University Hospital (PSCUH) were included in the study. Patients with MEN1 syndrome and mixed neuroendocrine and non-neuroendocrine neoplasms were excluded.

Preoperative, operative, and postoperative data from medical files and electronic systems were retrospectively collected in both hospitals by trained physicians. The data included patient demographics, clinical presentations, intraoperative variables (type of surgical approach), postoperative hospital stay, morbidity and mortality (within 30 days from surgery), pathological findings, and long-term follow-up (time to recurrence). Since 2016, data from both centers have been stored in EUROCRINE, an open-label international endocrine surgical registry with a special focus on rare tumors, which is an online platform.

Histological grade and differentiation were classified using the World Health Organization (WHO) 2017 criteria (18). Classification was based on the mitotic rate per 10 high-power fields (HPF) and the Ki-67 index in immunochemistry as: G1 tumors, mitotic count <2 per 10 HPF and Ki-67 ≤ 3%; G2 tumors, mitotic count 2–20 per 10 HPF and Ki-67 3%–20%; G3 tumors, mitotic count >20 per 10 HPF and Ki-67 > 20%. Based on morphology, G3 tumors were subcategorized into: well differentiated NEN G3 tumors and poorly differentiated NEC G3 tumors. Consequently, the PNEN stages were classified according to the TNM classification based on the 8th edition of the American Joint Committee on Cancer/Union for International Cancer Control (AJCC/UICC) staging criteria. If the initial grade or stage could not be assessed after data review, then, these tumors were included as “unknown”.

### 2.2. Analysis of outcomes

Postoperative complications that occurred in the first 30 day after surgery were defined by the Clavien–Dindo grading scale. Grade I and II complications were considered to be minor complications and Grades III to V were considered to be major complications.

Follow-up data were obtained from the outpatient care unit or oncological follow-up visits until August 2021. The follow-up parameters included information of oncological treatment modalities, current state of disease, and in case of death the cause of death. The Latvian Center for Disease Prevention and Control was consulted to identify if the included patients were still alive. Overall survival (OS) was defined as the time from diagnosis to death from any cause or, in living patients, the time to the last follow-up. Recurrence of disease was defined as evidence of any suspicious lesion found on imaging with or without tissue biopsy.

### 2.3. Statistical analysis

Descriptive statistics were used to summarize clinicopathological parameters and were expressed as the median value with interquartile range. Categorical variables were reported as numbers and percentages. Survival duration was measured using the Kaplan–Meier method and compared using a log rank test. A Cox's regression analysis was performed to identify factors independently associated with prognosis. The multivariate analysis included clinically important parameters identified on a univariate analysis. Hazard ratios (HR) and 95% confidence intervals (CIs) were presented for all variables. A value of *p* < 0.05 was considered to be statistically significant.

All the statistical analyses were performed using MS Excel, IBM SPSS Statistics version 29.0 for Windows.

## 3. Results

### 3.1. Patients and tumor characteristics

**Figure 1:**
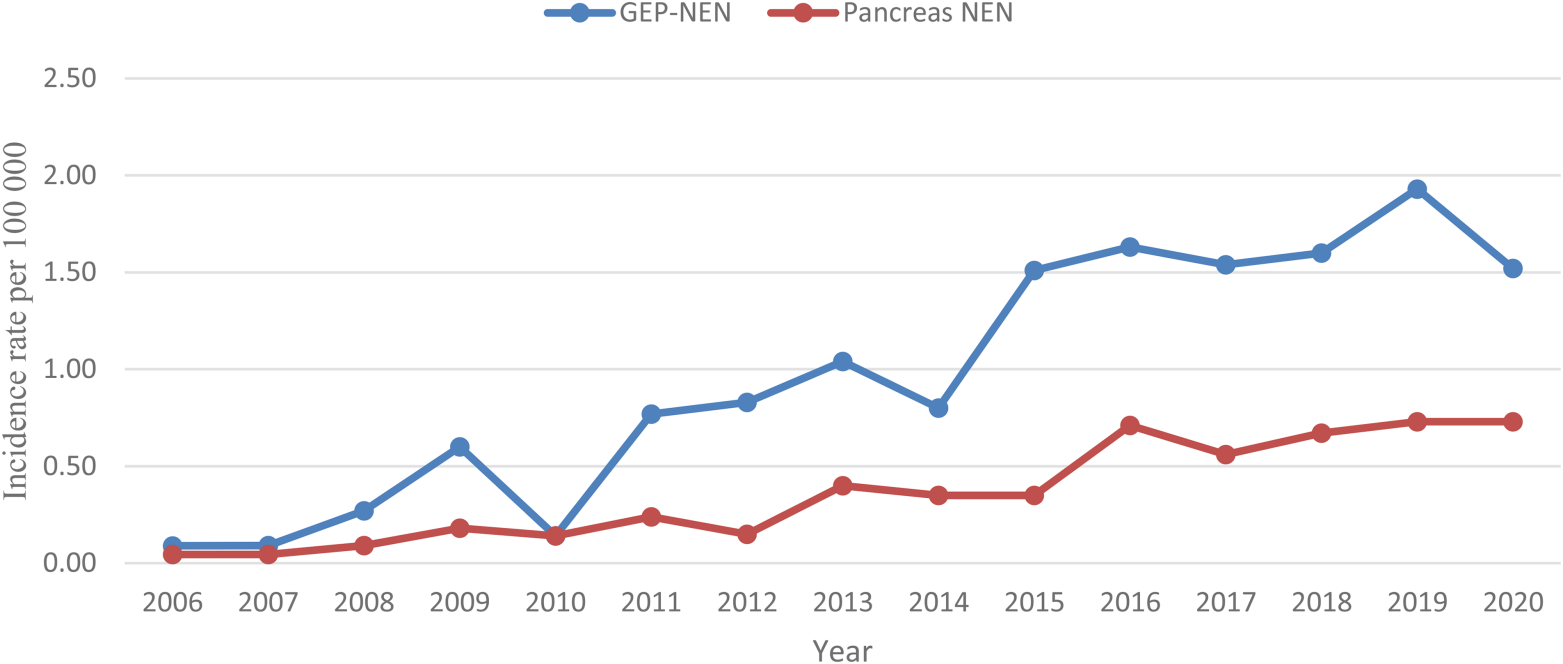
Age-standartized incidence rate of GEP-NEN and PNEN per 100,000 persons.

During the study period from January 2008 to December 2020, 105 patients with PNEN were treated at RECUH and PSCUH. As shown in Figure 1, incidence rate for PNEN in the population of Latvia from 2008 to 2020 increased from 0.09 to 0.73 per 100 000. Overall, 66 patients (62.9%) were female. The median age at diagnosis was 64 years (IQR 53.0–70.0) for men and 61 years (IQR 52.5–69.0) for women.

The disease was incidentally diagnosed in 31 patients (29.5%) with no symptoms on a visit to the hospital. Furthermore, symptoms associated with hypersecretion of bioactive substances were detected in 24 patients (22.9%). Among the known functional status, 11 patients (10.5%) presented with hypoglycemia and were diagnosed with insulinoma. Seven patients (6.7%) of patients presented with symptoms related to carcinoid syndrome.

Overall, in 37 patients (35.2%), primary tumors were located in the tail of the pancreas; head, neck, body, and overlapping accounted for 34 (32.4%), 1 (1.0%), 24 (22.9%), and 9 (8.6%) cases, respectively. Of note, 32 patients (30.5%) showed distant metastases at the time of diagnosis. Most of these metastases (23/32) were found in the liver, and six (5.7%) of these patients also showed synchronous lesions in bones and lungs; peritoneum metastases were confirmed in three (2.9%). In our study population we did not observed metastatic insulinomas. The detailed baseline characteristics of the studied population are summarized in Table 1.

**Table 1:** Baseline characteristics of included patients, tumors, and symptoms.

Characteristics	All patients	Nf-PNEN	F-PNEN
	*n* (%)	*n (%)*	*n (%)*
	*n *= 105	*n = 81*	*n = 24*
Age (years)
Median (IQR)	62 (53–69)	62 (51.5-69.0)	63.5 (55.8-69.0)
Gender
Male	39 (37.1)	36 (44.4)	3 (12.5)
Female	66 (62.9)	45 (55.6)	21 (87.5)
Symptoms
Asymptomatic	31 (29.5)	31 (38.3)	0
Site of pancreatic NEN
Head	34 (32.4)	26 (32.9)	8 (33.3)
Neck	1 (1.0)	1 (1.3)	0
Body	24 (22.9)	18 (22.8)	6 (25.0)
Tail	37 (35.2)	28 (35.4)	9 (37.5)
Overlapping	9 (8.6)	8 (9.9)	1 (4.2)
Disease spread
With distant MTS	32 (30.5)	25 (32.1)	7 (29.2)
MTS localization
Liver MTS only	23 (21.9)	17 (22.4)	6 (26.1)
Widespread MTS (multiple sites)	6 (5.7)	5 (6.6)	1 (4.3)
Other localization MTS	3 (2.9)	3 (3.9)	0

Abbreviations: MTS, metastases; Nf-PNEN, nonfunctional PNEN; F-PNEN, functional PNEN.

### 3.2. Treatment

Generally, patients without evidence of distant metastases underwent oncological resection with or without regional lymphadenectomy as a first-line therapy. Based on tumor functionality, stage, and location, 18 patients (17.1%) underwent pancreaticoduodenectomy, 33 patients (31.4%) had distal pancreatectomy, and 10 patients (9.5%) underwent enucleation. All of the enucleations were performed for insulinomas. Moreover, one patient (1.4%) underwent total pancreatectomy due to multifocal disease, while three patients (2.9%) underwent simultaneous liver resections for metastatic disease. Loco-regional therapy such as transcatheter hepatic arterial chemoembolization for the treatment of liver metastases was carried out in three patients. All the resections were performed in an elective schedule. Table 2 summarizes the treatment data.

**Table 2:** Treatment modalities for the PNEN patients.

Variables	All patients, *n* (%)	Non-metastatic PNEN *n* = 73	Metastatic PNEN *n* = 32
Surgery	71 (67.6)	62 (84.9)	9 (28.1)
Without surgery	34 (32.4)	10 (13.7)	23 (71.9)
Type of resection
Enucleation	10 (9.5)	10 (13.7)	0
DP without splenectomy	21 (20.0)	19 (26.0)	2 (6.3)
DP with splenectomy	12 (11.4)	9 (12.3)	3 (9.4)
PD	18 (17.1)	17 (23.3)	1 (3.1)
TP	1 (1.4)	1 (1.4)	0
Other unspecified	9 (8.6)	6 (8.2)	3 (9.4)
Chemotherapy
Yes	17 (16.2)	2 (2.7)	15 (46.9)
Radiotherapy
Yes	2 (1.9)	0	2 (6.3)
Somatostatin analogue
Yes	13 (12.4)	4 (5.5)	9 (28.1)

Abbreviations: PD, pancreaticoduodenectomy; DP, distal pancreatectomy; TP, total pancreatectomy.

Preoperative biopsies either from the primary tumor or a metastatic lesion histologically confirmed the diagnosis in 18.3% (13/71) of patients for a preoperative diagnostic accuracy of 100%. In all other PNEN patients, diagnosis was confirmed only after surgery.

Moreover, two patients (1.9%) received adjuvant treatment and 15 patients (14.3%) were treated with palliative chemotherapy. All regimens for chemotherapy were recommended by MDT assessments. Thirteen patients (12.4%) received biological therapy with somatostatin analogues.

Furthermore, starting from year 2017 for five patients (4.8%) with nonfunctional PNEN <2 cm, we used a “watch and wait” approach with careful monitoring of tumor size. Of note, none of the patients who were followed radiographically developed metastatic disease or progressed beyond resectability during the surveillance period.

### 3.3. Pathology

Neuroendocrine differentiation was confirmed by the immunohistochemical expression of chromogranin A (*n* = 69), synaptophysin (*n* = 69), or both (*n* = 60). Complete data for grading and staging were available for 90 (85.7%) and 96 patients (91.4%), respectively. According to the pathology reports, 40 patients (38.1%) had G1 tumors and 39 patients (37.1%) had G2 tumors, while four patients (3.8%) and seven patients (6.7%) had NEN G3 and NEC G3 tumors, respectively. According to the TNM classification, 39 tumors (37.1%) were in Stage I, 14 tumors (13.3%) were in Stage II, 12 tumors (11.4%) were in Stage III, and 32 tumors (30.5%) were in Stage IV. The histopathological data are provided in Table 3.

**Table 3:** Pathological characteristics of PNEN.

Characteristics	*n* (%)
WHO classification
G1	40 (38.1)
G2	39 (37.1)
NEN G3	4 (3.8)
NEC G3	7 (6.7)
Unknown	15 (14.3)
Tumor stage (TNM)
Stage I	39 (37.1)
Stage II	14 (13.3)
Stage III	12 (11.4)
Stage IV	32 (30.5)
Unknown	8 (7.6)
Neoplasm size on histopathology (cm)
<1 cm	5 (4.8)
1–2 cm	35 (33.3)
2–3 cm	18 (17.1)
>3 cm	36 (34.3)
No input	11 (10.5)
Negative surgical margin (R0)	64 (90.1)
Resected lymph node (LN) status
Positive LN	10 (14.1)
Immunohistochemical staining
Positive CgA	69 (79.3)
Positive Syn	69 (77.5)
Positive CgA and Syn	60 (70.6)

CgA, chromogranin A; Syn, synaptophysin.

Regarding resected PNEN, on the final histology, R0 resection was achieved in 64 patients (90.1%), and 10 patients (14.1%) had at least one lymph node metastases. Two patients who had R1 resection developed recurrence after surgery.

### 3.4. Postoperative outcomes

The median length of hospital stay was 8 days (IQR 5–12.5). Postoperative complications occurred in 15 patients (21.1%). Major postoperative complications were found in five patients (7.0%), and reoperation was performed for three patients (4.2%) due to postpancreatectomy bleeding (2/71) and abdominal collection (1/71). The detailed postoperative data are provided in Table 4.

**Table 4:** Postoperative outcomes.

Characteristics	*n* (%)
Hospital stay, days (median, IQR)	8 (5–12.5)
Overall complications	15 (21.1)
Minor complications (Grade I, II)	10 (14.1)
Major complications (≧ Grade III)	5 (7.0)
Pancreatic fistula	10 (14.1)
Reoperation	3 (4.2)
Mortality	2 (2.8)

### 3.5. Follow-up

**Figure 2:**
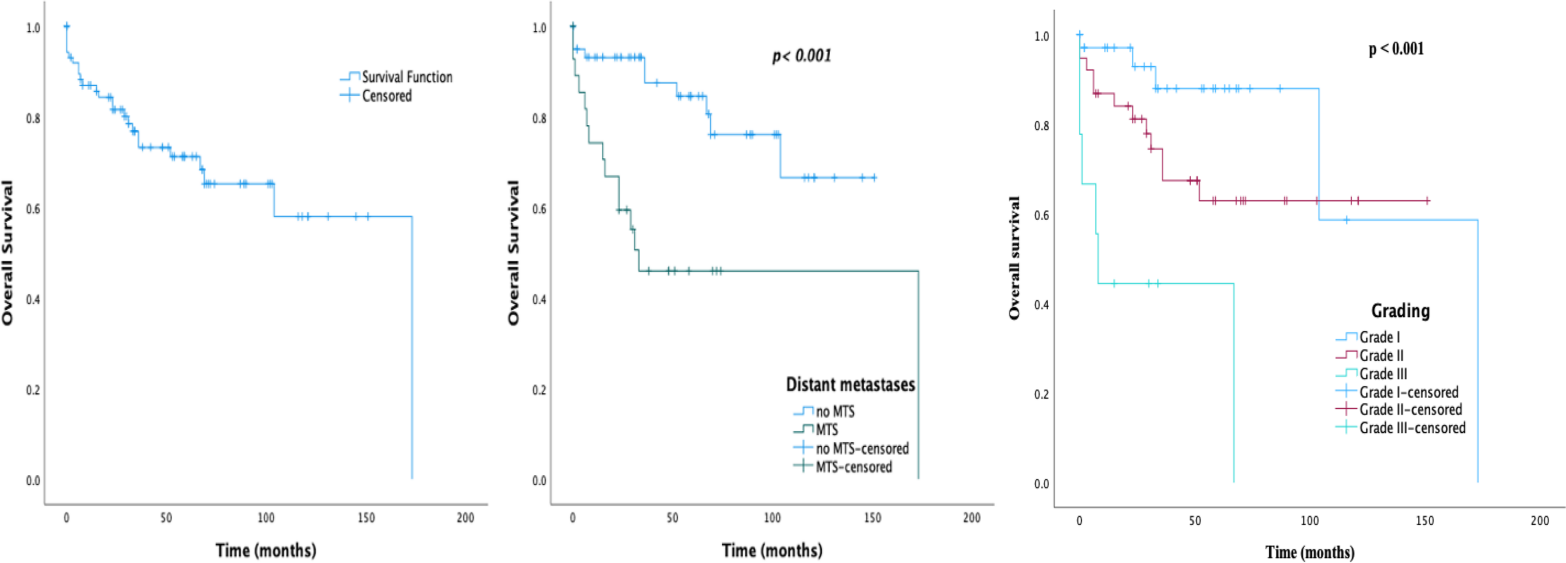
Survival graphs on 105 patients with sporadic PNEN diagnosis: (**A**) overall survival; (**B**) overall survival by MTS presentation; (**C**) overall survival by grade.

The median follow-up period was 34 months (IQR 15.0–68.8). Among the total 105 patients, 23.8% (25/105) died by the end of follow up. The observed 1-, 5-, and 10-year survival rates were 87.0%, 71.2%, and 58.0%, respectively. As shown in Figure 2, patients with metastases and a higher grading had poorer survival rates as compared with patients without (*p *< 0.001). The 1-, 5-, and 10-year survival rates of patients without distant metastases were 93.2%, 84.6%, and 66.6%, respectively. Of note, seven of the surgically treated patients (9.9%) had tumor recurrence. The median time of recurrence was 39 months (IQR 19.0–95.0).

The prognostic factors of OS are shown in Table 5. On the univariate analysis, nonfunctional tumor, presence of distant metastases, tumor size, and increasing tumor grade and stage were associated with significantly lower survival. The variables *p *< 0.1 in the Cox univariate analysis were further analyzed using multivariate analysis. After the multivariate Cox regression analysis NEC G3 was an independent factor that increased the risk of death.

**Table 5:** Univariate and multivariable Cox proportional hazards regression model for OS.

Characteristics	Univariate		Multivariate	
	HR (95% CI)	*p*-value	HR (95% CI)	*p*-value
Gender
Male	reference			
Female	0.62 (0.27–1.42)	0.257		
Functionality
Functional	reference		reference	
Nonfunctional	2.02 (0.88–4.63)	0.096	1.91 (0.68–5.39)	0.220
Tu localisation
Head	reference			
Body	0.97 (0.34–2.75)	0.951		
Tail	0.65 (0.24–1.74)	0.385		
Overlapping	2.59 (0.56–12.08)	0.225		
Distant metastases
No	reference		reference	
Yes	3.99 (1.75–9.12)	0.001	4.59 (0.57–37.16)	0.153
Tumor size
<1 cm	reference		reference	
1–2 cm			NA	
2–3 cm	0.16 (0.05–0.54)	0.003	NA	
>3 cm	0.56 (0.20–1.54)	0.261	0.79 (0.23–2.68)	0.704
Resected LN status
Positive LN	2.22 (0.81-6.06)	0.120		
Grade
G1	reference		reference	
G2	2.41 (0.78–7.47)	0.128	2.42 (0.53–11.15)	0.256
NEN G3	3.59 (0.40–32.30)	0.255	2.89 (0.23–36.83)	0.413
NEC G3	14.20 (3.60–56.02)	<0.001	6.21 (1.03–37.47)	0.042
Stage
Stage I	reference		reference	
Stage II	5.79 (0.52–63.87)	0.152	1.46 (0.12–18.20)	0.771
Stage III	19.33 (2.14–174.72)	0.008	3.21 (0.32–32.54)	0.325
Stage IV	23.63 (3.08–181.11)	0.002	NA	

NA, no value is available.

Note: Variables with univariate analysis *p* < 0.1 underwent further multivariate analysis.

## 4. Discussion

The rising trend in incidence of PNEN has attracted increased attention from researchers worldwide. Epidemiological studies have shown that PNEN account for approximately 1%–5% of all pancreatic neoplasms and, in turn, autopsy studies have shown that the prevalence may be as high as 10% (19–21). The are two main reasons regarding the rising incidence, i.e., improvement of available diagnostic techniques and clinicians awareness.

The current literature has reported that the prevalence of PNEN is higher in males worldwide (22). However, in our study, the prevalence of PNEN was higher in females than in males. In our cohort, the median age at diagnosis was 62 years, which was similar to previously reported studies from China and the SEER database (23, 24). Interestingly, Mengqi, L. et al. reported that the average age at diagnosis of PNEN was 52.6 ± 12.6 years, which was younger than we demonstrated (25).

Depending on the presence or absence of hormonal symptoms, PNEN can be classified as functioning or nonfunctioning. Due to their ability to produce hormones and subsequent hormonal symptoms, they are likely to be detected early. Nonfunctional PNEN may also secrete hormones at the subclinical level, causing nonspecific symptoms and often found incidentally or at diagnosis presenting as large primary tumors or advanced disease. In the literature, nonfunctional PNEN account for approximately 60%–90% of all PNEN (1, 19). As expected, our study also showed a trend towards an increase in the number of incidentally discovered PNEN. Notably, the proportion of carcinoid syndrome in patients with PNEN in previous case series or literature reviews was approximately 1%, while we observed it in 6.7% of patients with newly diagnosed PNEN (24, 26, 27).

Unequivocally, liver is the most common metastatic site for PNEN. Previous studies have revealed that from 60 to 90% of patients with neuroendocrine neoplasms develop liver metastasis during the course of disease (25). Overall, in our cohort, 30.5% of the PNEN showed distant metastases at diagnosis, with liver metastases in 71.9% of the PNEN. In addition, longer survival benefit was detected in patients without distant metastasis.

The diagnostic work-up for PNEN are same as those for pancreatic adenocarcinoma; including at least one high quality imaging examination (CT and/or MRI). For definitive diagnosis, immunohistochemical staining of tumor biopsy is mandatory. In our study, correct diagnosis of PNEN was confirmed preoperatively by EUS guided biopsy in 13 patients with diagnostic accuracy equal to 100%. As reported previously, the preoperative diagnostic accuracy of PNEN grading moderately to strongly correlated with surgical histology (28, 29). Although, our study showed excellent diagnostic accuracy rate, the results can not be considered reliable due to the small number of performed preoperative biopsies.

Historically, surgery has been the only optimal approach with curative intent of PNEN when complete resection is feasible for localized and symptomatic PNEN. The indications for surgery depend on tumor size, staging, the existence of genetic syndrome and comorbidities; additionally, for some cases (non-secreting tumors < 2 cm), the expected benefit rate vs. high surgical risk (for pancreatic surgery) must be carefully evaluated. To date, due to the rarity of these tumors, surgical management vs. active “surveillance” for small nonfunctional PNEN has been a hot topic of debate among pancreatic surgeons. Of note, for five patients with nonfunctional PNEN <2 cm, a “watch and wait” approach was used and none of the patients developed metastatic disease.

In the literature, the overall mortality in patients after surgical resection of pancreas has ranged between 2% and 6% in high volume hospitals (27). In our study, the overall in-hospital mortality was 2.8%.

For poorly differentiated PNEN or even for those with localized disease, only surgical treatment is not sufficient, medical treatments should also be applied. According to guidelines, medical treatment may include chemotherapy, biotherapy, targeting agents, and peptide receptor radionuclide therapy (13, 30).

Our study showed that 1-, 5- and 10-year OS was 87.0%, 71.2% and 58.0%, respectively. In agreement with other publications, OS in our study was compromised for patients with tumors that were in Stage IV. Since many studies have reported various parameters as prognostic factors for OS in patients with PNEN, we performed statistical analysis based on the most commonly described variables (gender, grade, stage, presentation of metastases, and tumor size) (1, 15–17). Based on the univariate analysis, the most important factors significantly affecting OS were larger tumor size, nonfunctional tumor, higher tumor grade and stage, and presentation of distant metastases, but after the multivariate analysis only NEC G3 was an independent risk factor associated with poor OS.

We acknowledge some limitations in our study. First, it is limited by the retrospective design, therefore, selection bias could have occurred. Second, some patients had incomplete basic information. However, to the best of our knowledge, to date, this retrospective analysis is one of the largest on PNEN in Baltic states, reporting detailed data regarding the clinical presentation, tumor characteristics, and treatment. Studies with larger sample sizes and multicenter data collection clarifying the clinical characteristics and survival associations may be of great value in managing PNEN.

## 5. Conclusions

Our study represents the general trends of clinicopathological features and treatment of PNEN in Latvia. For PNEN patients, tumor functionality, size, distant metastases, grade, and stage may be useful to predict OS and must be confirmed in further studies. Further, a “surveillance” strategy might be safe for selected patients with small asymptomatic PNEN.

## Data Availability

The raw data supporting the conclusions of this article will be made available by the authors, without undue reservation.
